# The prices outpatients have to pay for purchasing medicines in Armenia

**DOI:** 10.1186/2052-3211-8-S1-P20

**Published:** 2015-10-05

**Authors:** Irina Kazaryan, Anahit Sevikyan, Margarit Melikyan

**Affiliations:** 1 Department of Pharmaceutical Management, Yerevan State Medical University, Yerevan, 0025, Armenia; 2 Drug Utilization Research Group, Yerevan, 0019, Armenia

## Background

The great majority of patients in low- and middle-income countries purchase medicines out of pocket. Correspondingly, pharmaceuticals, especially those which are high cost, can be unaffordable for patients. Measuring medicine availability and prices is especially important for countries like Armenia, which are still not implementing policy for managing prices of pharmaceuticals. The objective of this study was evaluating the prices and availability of medicines in pharmacy outlets in Armenia.

## Methods

Data on availability and patient prices were collected in May 2015 from 30 private pharmacies and pharmacy kiosks in Yerevan, the capital of Armenia. for 50 medicines (with specific strength and dosage form) authorized in Armenia. For each medicine, all pf the products registered in the state were included in the survey form. In addition, data on the same medicines were collected from pricelists of the four main wholesalers. Patient prices were compared with International reference prices (IRP) to identify a median price ratio (MPR).

## Results

All of the medicines selected except one (Nicotine Gum) were listed in the wholesalers' pricelists and available in pharmacy outlets. The originator brands (OBs) for 21 and generics for 45 of 50 medicines studied were available on the pharmaceutical market. For four medicines (Perindopril, Valproic acid, Ipratropium bromide, Flixotide) only the OBs were found. For other four pharmaceuticals (Amlodipine, Omeprazole, Fluconazole, Ceftriaxone) more than 10 generics were available on the pharmaceutical market. Ten of 45 (22.2%) lowest priced generics (LPGs) were locally produced pharmaceuticals. OBs for 15 of 17 medicines were higher priced than LPGs. Using matched medicine pairs (OBs:LPGs ratio for 17 medicines), OBs were on average 9.8 times the price of LPGs. Five OBs were more than 10 times more expensive than the LPGs – up to 35 times more for Ciprofloxacin and 31 times for Fluconazole. The median MPR of patient prices was 16.2 for OBs and 2.1 for LPGs. The MPR for 15 of 16 OBs and for 10 of 40 LPGs was 5 and higher.

## Conclusions

Lowest priced generic equivalents were more available than OBs, however, they were not found for all medicines. Overall, patient prices were high. OBs were many times more expensive than the lowest priced generics. This can compromise affordability of medicines for those outpatients who purchase medicines out-of-pocket. Introduction and implementation of pricing policy in Armenia would help to manage medicine prices.

